# Association between periodontal disease and Alzheimer's disease: umbrella review

**DOI:** 10.3389/fdmed.2025.1635200

**Published:** 2025-07-09

**Authors:** Heber Isac Arbildo-Vega, Fredy Hugo Cruzado-Oliva, Franz Tito Coronel-Zubiate, Rubén Aguirre-Ipenza, Joan Manuel Meza-Málaga, Sara Antonieta Luján-Valencia, Eduardo Luján-Urviola, Adriana Echevarria-Goche, Carlos Alberto Farje-Gallardo, Tania Belú Castillo-Cornock, Katherine Serquen-Olano, Tania Padilla-Cáceres, Luz Caballero-Apaza

**Affiliations:** ^1^ Faculty of Dentistry, Dentistry School, Universidad San Martin de Porres, Chiclayo, Peru; ^2^ Faculty of Human Medicine, Human Medicine School, Universidad San Martín de Porres, Chiclayo, Peru; ^3^ Faculty of Stomatology, Stomatology School, Universidad Nacional de Trujillo, Trujillo, Peru; ^4^ Faculty of Health Sciences, Stomatology School, Universidad Nacional Toribio Rodríguez de Mendoza de Amazonas, Chachapoyas, Peru; ^5^ Faculty of Health Sciences, Universidad Continental, Lima, Peru; ^6^ Faculty of Dentistry, Dentistry School, Universidad Católica de Santa María, Arequipa, Peru; ^7^ Faculty of Medicine, Medicine School, Universidad Católica de Santa María, Arequipa, Peru; ^8^ Postgraduate School, Universidad Católica de Santa María, Arequipa, Peru; ^9^ Faculty of Dentistry, Universidad Andina Néstor Cáceres Velásquez, Juliaca, Peru; ^10^ Department of Dentistry, Dentistry School, Universidad Norbert Wiener, Lima, Peru; ^11^ Faculty of Health Sciences, Stomatology School, Universidad Señor de Sipán, Chiclayo, Peru; ^12^ Department of General Dentistry, Dentistry School, Universidad del Altiplano, Puno, Peru; ^13^ Research Institute in Environmental Sciences, Health and Biodiversity - IICASB, Universidad del Altiplano, Puno, Peru; ^14^ Department of Nursing, School of Nursing, Universidad del Altiplano, Puno, Peru

**Keywords:** Alzheimer's disease, periodontal disease, tooth loss, periodontitis, review

## Abstract

**Background:**

Alzheimer's disease (AD) and periodontal disease (PD) are both chronic conditions with rising global prevalence. Emerging research suggests a possible link between PD-induced systemic inflammation and neurodegeneration observed in AD.

**Objective:**

Employ an umbrella review to look into the association between periodontal disease and Alzheimer's disease.

**Materials and methods:**

A comprehensive search was conducted until March 2025 across various electronic databases, including: PubMed, Cochrane Library, Scopus, SciELO, Web of Science, Google Scholar, ProQuest, and OpenGrey, and Dissertations and Theses. There were no time or language restrictions on the inclusion of systematic reviews (SR), with or without meta-analysis, as long as they looked at primary research that connected PD and AD. The following were excluded: preclinical and fundamental research, summaries, comments, case reports, protocols, personal opinions, letters, posters, literary or narrative reviews, fast reviews, intervention studies, and observational studies. The quality and general confidence of the included studies were evaluated using the AMSTAR-2 technique.

**Results:**

A total of 358 items were found following the first search. 16 articles were left for additional review after the selection criteria were applied. With odds ratios (OR) and risk ratios (RR) ranging from 1.67 to 2.17 and 1.11 to 2.26, respectively, 14 studies showed a correlation between PD and AD.

**Conclusions:**

Drawing from the findings and conclusions of the SR demonstrating a high overall confidence, it's established that PD and tooth loss exhibit an association with AD.

**Systematic Review Registration:**

Registered in the Open Science Framework (OSF): DOI 10.17605/OSF.IO/GS367.

## 1 Introduction

Chronic diseases are responsible for approximately 60% of annual deaths worldwide, according to the WHO (1). Risk factors include age, genetics, obesity, poor diet, smoking, and physical inactivity (2). Although temporary inflammation is expected in the face of injury or infection, when local immune balance is not restored, chronic inflammation develops (3).

The progression of chronic diseases follows a common pattern: identification of the irritant, activation of inflammatory pathways, cytokine release, and cell recruitment (3, 4). The proinflammatory cytokines IL-1β, IL-6, and TNF-α, activate immune cells and perpetuate inflammation (5–7).

The human oral microbiota is composed of approximately 770 microorganisms that form a biofilm on the teeth (8). Under optimal hygiene conditions, a healthy balance of gram-positive bacteria predominates. However, factors such as high sugar intake, acid exposure, or poor brushing habits can alter composition (9). Diseases like periodontal disease (PD) can result from this imbalance, known as oral dysbiosis, which promotes the growth of anaerobic gram-negative bacteria (8). Between 20% and 50% of people worldwide suffer from PD, which includes periodontitis and gingivitis, making it one of the most prevalent chronic illnesses (10, 11). The advanced stage of periodontitis is typified by tooth loss, loss of bone support, and ongoing inflammation (12, 13). It is caused by polymicrobial infections and poor oral hygiene habits, such as lack of flossing (8, 14).

When the immune system cannot completely eliminate oral bacteria, gum inflammation becomes chronic (13). This leads to a continuous release of destructive inflammatory cells. In this context, *Porphyromonas gingivalis*, *Treponema denticola*, *Fusubacterium nucleatum*, and *Aggregatibacter actinomycetemcomitans* are commonly associated with PD (8, 12, 15). Since they are also present in healthy mouths, it's clear that the host´s inflammatory response-rather than just the presence of bacteria- determines how a disease develops (5, 13, 16).

Alzheimer's disease (AD) affects 47 million people. AD is a long-term neuroinflammatory condition that causes brain degeneration, with the hippocampus being one of the most affected regions (17), cognitive decline, and eventually death (1, 8). Approximately 7 out of ten cases of dementia are related to this disease, moreover, its prevalence increases with age (1). By 2040, dementia is expected to affect more than 81 million people worldwide (18).

AD progresses through the preclinical stage, then becomes mild cognitive impairment and finally triggers severe dementia (1). In the first stage, there are no visible symptoms, but neuropathological changes and inflammatory markers are already present (19). These changes can begin decades before the onset of clinical symptoms. Currently, definitive diagnosis is made postmortem through a brain autopsy, which limits the possibilities for early intervention (1).

AD is marked by the accumulation of amyloid plaques alongside tau protein neurofibrillary tangles (20, 21). Although the amyloid hypothesis postulates that amyloid initiates brain damage, some studies show its presence in people without AD (22) and its absence in patients with clinical symptoms (21). In contrast, the tau hypothesis argues that this protein is present even in early stages without amyloid, and may be the primary contributor to neurodegeneration (23).

Moreover, to amyloid plaques and tau tangles, brain inflammation is also observed in AD. The inflammatory hypothesis argues that AD develops due to a host immune response caused by plaques and tangles, which activate immune cells such as microglia (24). This triggers the release of proinflammatory cytokines such as IL-1, IL-6, and TNF-α (25, 26), which in turn can generate more amyloid and tau (27). As in PD, the interaction between bacteria and host immunity determines the severity of brain damage (1).

According to the focal infection idea, localized infections are the root cause of many chronic illnesses (27). From this perspective, a local alteration can trigger systemic effects, as microorganisms and inflammatory mediators can circulate throughout the body (1). Recently, a relationship has been identified between periodontitis and diseases such as atherosclerosis and diabetes mellitus, where oral bacteria can enter the bloodstream and promote systemic inflammation (10, 28–30).

Several studies show that periodontitis is associated with an increased risk of cardiovascular events, bacteremia, and systemic inflammation (10, 30–32). The relationship between diabetes mellitus (DM) and periodontitis has also been shown to be bidirectional: DM increases the risk of PD, and PD, in turn, impairs glycemic control (33).

Recent studies have investigated the potential influence of gut microbiota on neurodegenerative diseases, including Alzheimer's disease (14, 34). Stress-related disruptions of the gur-brain axis can elevate intestinal permeability, permitting endotoxins to breach the bloodstream. In turn, this elicits an immune reaction that may reach the central nervous system and foster disorders such as anxiety and depression (35), however, a direct causal link to Alzheimer's disease has yet to be established (14).

The Spanish Society of Neurology described a possible connection between periodontal disease and Alzheimer's disease. AD involves progressive brain degeneration, with loss of memory and cognitive skills, making independent living difficult. Furthermore, the relationship may be bidirectional: cognitive decline in AD reduces oral hygiene, increasing periodontal inflammation, while chronic periodontal infection may contribute to neuroinflammation and accelerate cognitive deterioration (14, 36, 37). This interplay highlights the importance of oral health maintenance in elderly patients with cognitive decline, especially considering the increasing susceptibility to infection and reduced ability to perform adequate self-care.

According to one study, Alzheimer's disease patients tend to have more missing teeth and edentulism than people without the disease (38). This suggests that tooth loss, potentially related to PD, may be implicated in the development of AD. A recent finding highlights the involvement of *Porphyromonas gingivalis*, a pathogenic bacterium linked to PD, in cognitive decline due to the release of inflammatory toxins (8, 39–43).

Increasing evidence has shown that *Porphyromonas gingivalis*, a keystone pathogen in periodontitis, can invade the brain, promoting neuroinflammation and tau protein phosphorylation through its virulence factor gingipains (44, 45). In murine models, oral infection with *Porphyromonas gingivalis* resulted in brain colonization and increased amyloid-β production, supporting a causal role (44). Moreover, *Porphyromonas gingivalis* DNA and gingipains have been detected in the hippocampus of AD patients (45), suggesting a potential mechanistic pathway between oral infection and neurodegeneration.

Research suggests a bidirectional relationship between PD and AD: PD may increase the risk of Alzheimer's disease and vice versa (46, 47). In accordance to research by Qi et al., the risk of dementia rose by 1.1% and the chance of cognitive impairment by 1.4% for every tooth lost (37). Although PD is the leading cause of tooth loss (1), how this loss is linked to cognitive decline is not yet fully understood. Two pathways have been proposed: oral bacterial migration to other organs and secondary systemic inflammation (4).

In recent years, scientific research has focused more attention on the relationship between oral health and neurodegenerative diseases. AD, one of the most prevalent conditions, continues to be studied to understand its complex etiology. In this context, PD, a chronic inflammatory oral disease, has gained prominence as a possible factor in the development or progression of AD (44). This growing interest promotes an interdisciplinary approach that integrates oral health as a fundamental component of neurological well-being.

**Figure 1:**
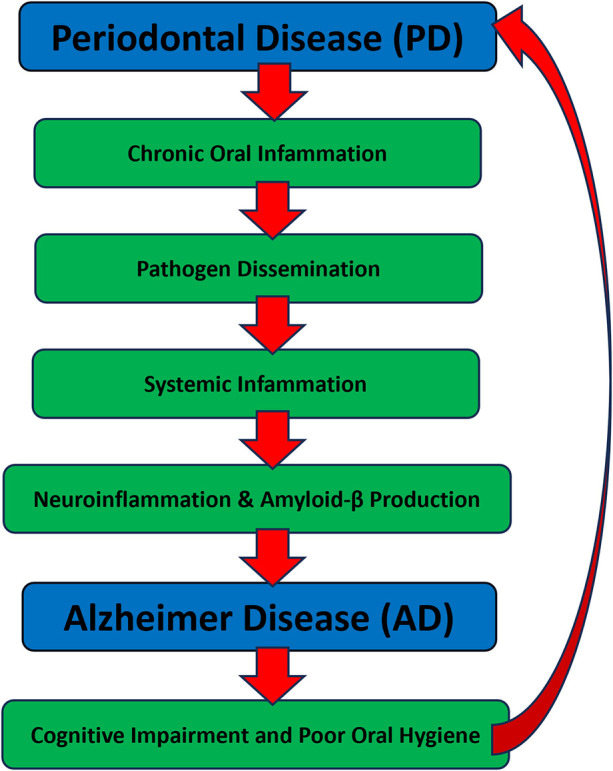
Hypothesized pathways linking periodontal disease and Alzheimeŕs disease. The diagram illustrates the proposed bidirectional relationship between periodontal disease (PD) and Alzheimeŕs disease (AD). Chronic inflammation originating in PD promotes pathogen dissemination and systemic immune activation, which can lead to neuroinflammation, amyloid-*β* accumulation, and Alzheimeŕs pathology. As AD progresses, cognitive decline and reduced self-care capacity impair oral hygiene, exacerbating periodontal inflammation. This creates a self-perpetuating cycle involving bacterial virulence factors (e.g., gingipains), proinflammatory cytokines (e.g., IL-1β, IL-6, TNF-α), and disruption of the blood-brain barrier.

Figure 1 illustrates the hypothesized biological pathways linking periodontal disease with Alzheimeŕs disease. This schematic representation integrates current evidence on microbial translocation, systemic inflammation, neuroimmune responses, and behavioral changes, and highlights the bidirectional interplay between both conditions.

Therefore, the objective of this overview was to consolidate the existing evidence and address the following question: “What is the current understanding regarding the relationship between PD and AD?” Additionally, how overall confidence are systematic reviews in evaluating this topic?

## 2 Materials and methods

### 2.1 Protocol and registration

We drafted a protocol aligned with the PRISMA-P guidelines (48) and officially registered it on the Open Science Framework (DOI 10.17605/OSF.IO/GS367). The review follows the PRIO-harms (49), and no ethical clearance was required for this umbrella review.

The research question was formulated using the PECO framework (population, exposure, comparison, and outcomes), aimed to evaluate the association between periodontal disease and Alzheimer's disease. The PECO elements were defined as follows:

– Population: individuals of all ages evaluated for cognition function.
– Exposure: individuals with periodontal disease or tooth loss.
– Comparison: individuals without periodontal disease.
– Outcomes: diagnosis or risk of Alzheimeŕs disease, cognitive impairment, or dementia.

This framework guided the elegibility criteria, for the included systematic reviews. Only reviews reporting a measurable association betweern PD and AD (or related cognitive outcomes) were considered.

### 2.2 Eligibility criteria and results of interest

The eligible studies comprised SR with or without meta-analysis, without limitations on publication date or language, that investigated primary studies exploring the association between PD and AD. Excluded were literature or narrative reviews, rapid reviews, intervention studies, observational studies, preclinical and basic research, abstracts, commentaries, case reports, protocols, personal opinions, letters, and posters.

### 2.3 Sources of information, search strategy, and additional search for primary studies

On March 25th, 2025, an electronic search was conducted across five databases, including PubMed, Cochrane database, Scielo, Web of Science, and Scopus. Grey literature was explored using the top 100 reports from Google Scholar, Proquest Dissertations and Theses, and OpenGrey. Furthermore, reference lists of the included studies were screened. Retrieved articles were managed using reference management software (Zotero® 6.0, Center for History and New Media, Fairfax, Virginia, USA), and duplicate entries were eliminated. The search strategies implemented for each database are detailed in Table 1.

**Table 1:** Search strategy for each search engine.

Database	Search strategy	Number of studies
Pubmed	(“Periodontal Diseases” OR “Periodontitis” OR “Periodontitides” OR “Chronic Periodontitis” OR “Aggressive Periodontitis” OR “Periodontal Disease” OR “Periodontal Infections”) AND (“Alzheimer Disease” OR “Alzheimer's Disease” OR “Disease, Alzheimer” OR “Alzheimer Dementia” OR “Dementia, Alzheimer” OR “Alzheimer Type Dementia” OR “Dementia, Alzheimer Type” OR “Alzheimer-Type Dementia” OR “Dementia, Alzheimer-Type” OR “Alzheimer-Type Dementia (ATD)” OR “Dementia, Alzheimer-Type (ATD)” OR “Alzheimer Type Senile Dementia” OR “Senile Dementia, Alzheimer Type” OR “Sclerosis, Alzheimer” OR “Alzheimer Syndrome” OR “Cognitive Impairment” OR “Cognitive Decline” OR “Cognitive Loss” OR “Poor Cognitive Function” OR “Cognitive dysfunction” OR “Presenile Alzheimer Dementia” OR “Presenile Dementia” OR “Dementia, Presenile” OR “Senile Dementia” OR “Dementia, Senile” OR “Neurodegenerative Diseases” OR “Neurodegenerative Disorders” OR “Neurological Degenerative Diseases” OR “Neurological Degenerative Conditions” OR “Nervous System Degenerative Diseases”) AND (“Systematic review” OR “Meta-analysis”)	58
Cochrane database	#1 MeSH descriptor: [Periodontal Diseases] explode all trees #2 MeSH descriptor: [Periodontitis] in all MeSH products #3 (“Periodontal Disease”):ti,ab,kw OR (“Periodontitis”):ti,ab,kw OR (“Chronic Periodontitis”):ti,ab,kw OR (“Aggressive Periodontitis”):ti,ab,kw OR (“Periodontal Infections”):ti,ab,kw (Word variations have been searched) #4 #1 OR #2 OR #3 #5 MeSH descriptor: [Alzheimer Disease] explode all trees #6 MeSH descriptor: [Cognitive Dysfunction] explode all trees #7 MeSH descriptor: [Neurodegenerative Diseases] explode all trees #8 (“Alzheimer Disease”):ti,ab,kw OR (“Alzheimer's Disease”):ti,ab,kw OR (“Alzheimer Dementia”):ti,ab,kw OR (“Sclerosis, Alzheimer”):ti,ab,kw OR (“Alzheimer Syndrome”):ti,ab,kw OR (“Cognitive Impairment”):ti,ab,kw OR (“Cognitive Decline”):ti,ab,kw OR (“Cognitive Loss”):ti,ab,kw OR (“Cognitive dysfunction”):ti,ab,kw OR (“Presenile Alzheimer Dementia”):ti,ab,kw OR (“Presenile Dementia”):ti,ab,kw OR (“Senile Dementia”):ti,ab,kw OR (“Neurodegenerative Diseases”):ti,ab,kw OR (“Neurodegenerative Disorders”):ti,ab,kw OR (“Neurological Degenerative Diseases”):ti,ab,kw OR (“Neurological Degenerative Conditions”):ti,ab,kw OR (“Nervous System Degenerative Diseases”):ti,ab,kw (Word variations have been searched) #9 #6 OR #7 OR #8 #10 MeSH descriptor: [Systematic Reviews as Topic] explode all trees #11 MeSH descriptor: [Meta-Analysis as Topic] explode all trees #12 #10 OR #11 #13 #4 AND #9 AND #12	0
Scielo	(“Periodontal Diseases” OR “Periodontitis” OR “Chronic Periodontitis” OR “Aggressive Periodontitis” OR “Periodontal Disease”) AND (“Alzheimer Disease” OR “Alzheimer's Disease” OR “Nervous System Diseases”) AND (“Systematic review” OR “Meta-analysis”)	0
Scopus	TITLE-ABS-KEY ((“Periodontal Diseases” OR “Periodontitis” OR “Periodontitides” OR “Chronic Periodontitis” OR “Aggressive Periodontitis” OR “Periodontal Disease” OR “Periodontal Infections”)) AND TITLE-ABS-KEY ((“Alzheimer Disease” OR “Alzheimer's Disease” OR “Disease, Alzheimer” OR “Alzheimer Dementia” OR “Dementia, Alzheimer” OR “Alzheimer Type Dementia” OR “Dementia, Alzheimer Type” OR “Alzheimer-Type Dementia” OR “Dementia, Alzheimer-Type” OR “Alzheimer-Type Dementia (ATD)” OR “Dementia, Alzheimer-Type (ATD)” OR “Alzheimer Type Senile Dementia” OR “Senile Dementia, Alzheimer Type” OR “Sclerosis, Alzheimer” OR “Alzheimer Syndrome” OR “Cognitive Impairment” OR “Cognitive Decline” OR “Cognitive Loss” OR “Poor Cognitive Function” OR “Cognitive dysfunction” OR “Presenile Alzheimer Dementia” OR “Presenile Dementia” OR “Dementia, Presenile” OR “Senile Dementia” OR “Dementia, Senile” OR “Neurodegenerative Diseases” OR “Neurodegenerative Disorders” OR “Neurological Degenerative Diseases” OR “Neurological Degenerative Conditions” OR “Nervous System Degenerative Diseases”)) AND TITLE-ABS-KEY ((“Systematic review” OR “Meta-analysis”))	114
Web of Science	TS = (“Periodontal Diseases” OR “Periodontitis” OR “Periodontitides” OR “Chronic Periodontitis” OR “Aggressive Periodontitis” OR “Periodontal Disease” OR “Periodontal Infections”) AND TS = (“Alzheimer Disease” OR “Alzheimer's Disease” OR “Disease, Alzheimer” OR “Alzheimer Dementia” OR “Dementia, Alzheimer” OR “Alzheimer Type Dementia” OR “Dementia, Alzheimer Type” OR “Alzheimer-Type Dementia” OR “Dementia, Alzheimer-Type” OR “Alzheimer-Type Dementia (ATD)” OR “Dementia, Alzheimer-Type (ATD)” OR “Alzheimer Type Senile Dementia” OR “Senile Dementia, Alzheimer Type” OR “Sclerosis, Alzheimer” OR “Alzheimer Syndrome” OR “Cognitive Impairment” OR “Cognitive Decline” OR “Cognitive Loss” OR “Poor Cognitive Function” OR “Cognitive dysfunction” OR “Presenile Alzheimer Dementia” OR “Presenile Dementia” OR “Dementia, Presenile” OR “Senile Dementia” OR “Dementia, Senile” OR “Neurodegenerative Diseases” OR “Neurodegenerative Disorders” OR “Neurological Degenerative Diseases” OR “Neurological Degenerative Conditions” OR “Nervous System Degenerative Diseases”) AND TS = (“Systematic review” OR “Meta-analysis”)	66
Google Scholar	“Periodontal disease” “Periodontitis” “Alzheimer Disease” “Alzheimer's Disease” “Systematic review” “Meta-analysis”	100
Proquest Dissertations and Theses	(“Periodontal Diseases” OR “Periodontitis” OR “Chronic Periodontitis” OR “Aggressive Periodontitis” OR “Periodontal Disease”) AND (“Alzheimer Disease” OR “Alzheimer's Disease” OR “Nervous System Diseases”) AND (“Systematic review” OR “Meta-analysis”) NOT (“Literature” OR “review”)	20
OpenGrey	(“Periodontal Diseases” OR “Periodontitis” OR “Chronic Periodontitis” OR “Aggressive Periodontitis” OR “Periodontal Disease”) AND (“Alzheimer Disease” OR “Alzheimer's Disease” OR “Nervous System Diseases”) AND (“Systematic review” OR “Meta-analysis”)	0

### 2.4 Data management and selection process

All identified articles were imported into Rayyan®, an online systematic review platform maintained by the Qatar Computing Research Institute in Doha, Qatar. The study Selection process was conducted in two sequential phases. In the first phase, two reviewers (F.C.O and F.C.Z) independently evaluated the titles and abstracts of the identified articles.

Subsequently, in the second phase, the same two reviewers independently reviewed the full text of each article. Any discrepancies between the two reviewers were resolved through consultation with a third reviewer (H.A.).

### 2.5 Data collection process

Data were independently and in duplicate extracted from each study using a standardized table prepared by two reviewers (F.C.O and R.A.). These entries were then cross-checked, and any disagreements were settled by consulting the third author (H.A.). The information gathered from the chosen articles comprised author names, publication year, study and primary designs, count of studies included in qualitative vs. quantitative synthesis, findings, key conclusions, and any mention of employed frameworks or methods- such as PRISMA, PROSPERO, GRADE, and meta-analysis.

### 2.6 Assessment of methodological quality, quality of evidence, and meta-bias

Two reviewers (J.M. and S.L.) independently conducted a duplicate evaluation of the methodological quality of the included SRs, with a calibration of Kappa 0.85, using the AMSTAR-2 checklist (A Measurement Tool to Assess Systemic Reviews) (50). AMSTAR-2 assesses the methodological quality of SRs through 16 questions, each with three possible responses: “yes,” “no,” or “partially yes.” The overall confidence rating of the studies, categorized as high, moderate, low, or critically low, was determined following the guidelines proposed by Shea et al. (50).

### 2.7 Summary of measures

For SR without meta-analysis, we considered the summarized results from the primary studies included. However, if the SR included a meta-analysis, we focused on the results presented with odds ratio (OR) or risk/rate ratio (RR) to assess the association between PD and AD.

### 2.8 Summary of results

The primary outcomes of the included SR were summarized, with their findings classified into multiple AD-related categories.

## 3 Results

### 3.1 Review and selection of primary studies

**Figure 2:**
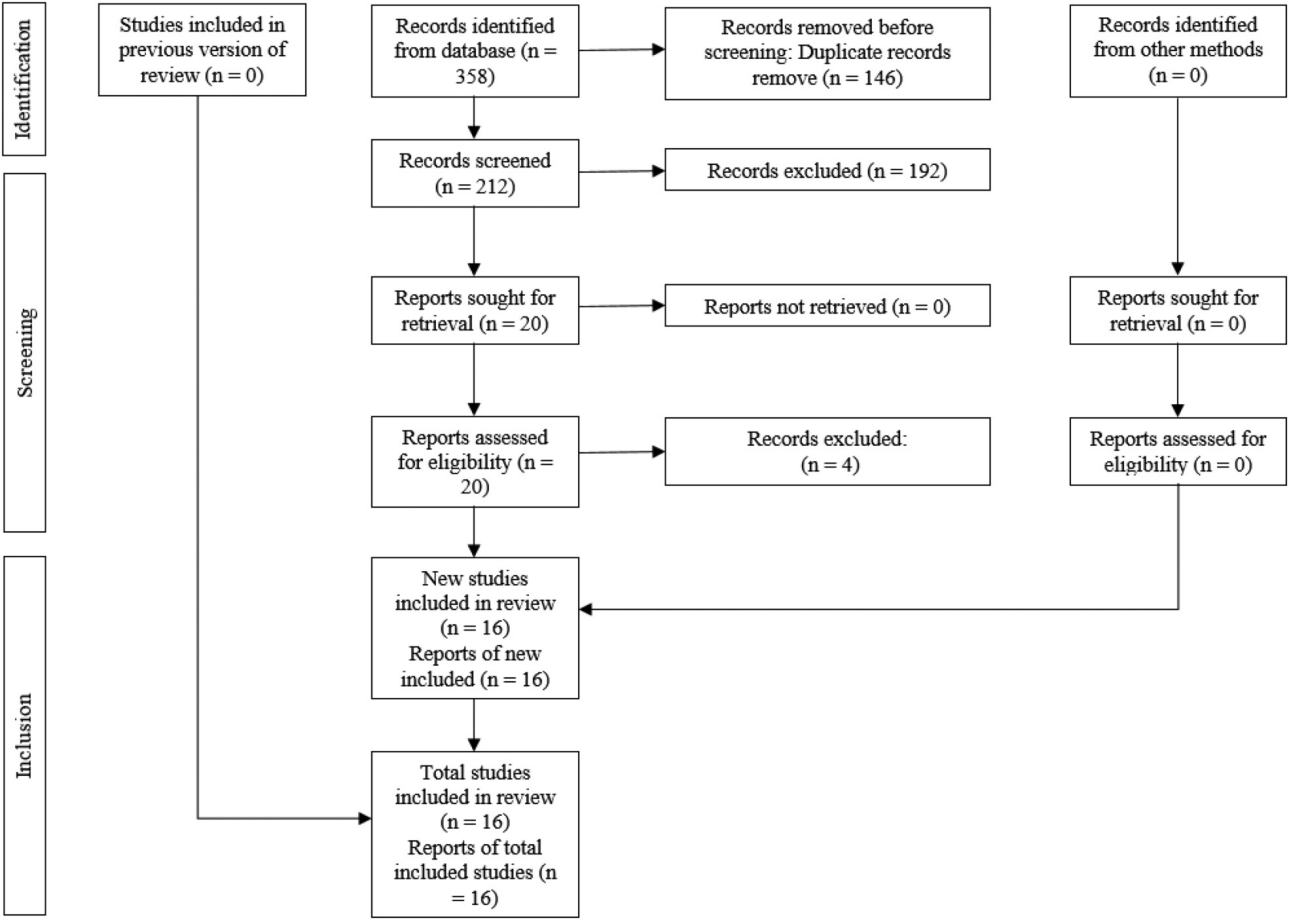
PRISMA flow diagram of the study selection process.

From the electronic database search, 358 articles, and 121 remained after duplicate removal. During phase one, titles and abstracts narrowing the list to 20 articles full-text review. Ultimately, 16 systematic reviews met inclusion criteria for qualitative synthesis. Article exclusion criteria appear in Table 2, and the complete study selection workflow is depicted in Figure 2.

**Table 2:** Reason for exclusion of studies.

Author	Reason for exclusion
Raymundo et al. (48)	No AD
Alvarenga et al. (49)	
Nascimento et al. (50)	
Lauritano et al. (51)	Association between PD and dementia

### 3.2 Review and characteristics of included studies

The SR included in the analysis were published between 2017 and 2025 and originated from various countries, including China (41, 52–54), France (8), India (55), Italy (38), Morocco (56), Poland (57), Republic of Korea (58), Singapore (59), Spain (60, 61), United Arab Emirates (62) and United Kingdom (63, 64). Thirteen (8, 41, 54–64) and three (38, 52, 53) SR evaluated the association of PD and tooth loss, respectively. Additional details pertaining to the characteristics of these systematic reviews can be found in Table 3.

**Table 3:** Characteristics of included studies.

Authors	Year	Study Design	Country	Included Study Design	Number of Studies in the Qualitative Analysis	Number of Studies in the Quantitative Analysis	Outcomes		Conclusions
Kim et al. (55)	2025	SR and MA	Republic of Korea	C and CC	24	24	Dementia	OR = 2.26 (1.65–3.09)	We found a strong association between periodontitis and dementia, with severe periodontitis identified as a potential risk factor for Alzheimer's dementia.
								HR = 1.15 (1.04–1.27)	
							Non—specified dementia	OR = 2.30 (1.25–4.23)	
							AD	OR = 2.17 (1.22–3.87)	
							Cognitive impairment	OR = 2.42 (1.49–3.92)	
							Dementia—Severe PD	OR = 2.85 (2.16–3.74)	
							Dementia—Moderate PD	OR = 0.94 (0.64–1.40)	
							Non—specified dementia—Severe PD	OR = 1.38 (0.63–3.03)	
							Non—specified dementia—Moderate PD	OR = 1.07 (0.66–1.73)	
							AD—Severe PD	OR = 6.87 (2.55–18.54)	
							AD—Moderate PD	OR = 0.73 (0.37–1.44)	
							Cognitive impairment—Severe PD	OR = 2.92 (2.15–3.96)	
Melo et al. (57)	2025	SR	Spain	CS	5	0	AD	Four studies supported connection between AD and periodontal inflammatory parameters, one study found no plausible association.	Most studies suggest a positive correlation between PD and AD.
Fu et al. (65)	2024	SR and MA	China	C and CC	22	22	Cognitive impairment—PD	OR = 1.45 (1.20–1.76)	Poor periodontal health, assessed across four dimensions (periodontitis, tooth loss, occlusal support, and masticatory ability), represents a significant risk factor for cognitive impairment in older adults. No significant association was found between tooth loss and the risk of developing AD.
							Cognitive impairment—tooth loss	OR = 1.80 (1.50–2.15)	
							Cognitive impairment—occlussal suport	OR = 1.87 (1.29–2.70)	
							Cognitive impairment—masticatory ability	OR = 1.39 (1.11–1.75)	
							Mild cognitive impairment—tooth loss	OR = 1.66 (1.43–1.91)	
							AD—tooth loss	OR = 3.89 (0.68–22.31)	
							Dementia—tooth loss	OR = 1.35 (1.11–1.65)	
Bouziane et al. (53)	2023	SR	Morocco	C and CC	6	0	AD	All studies except one showed a significant association between PD and the risk of AD onset and progression.	Patients with PD present a significantly higher risk of AD compared to individuals with healthy periodontium.
Ab Malik et al. (60)	2023	SR	United Kingdom	CC	10	0	Dementia and AD	A higher prevalence of subjects with severe periodontal disease was observed in individuals diagnosed with dementia/AD.	Although periodontitis is suggested as one of the risk factors for dementia and AD, the association remains unclear.
Nascimento et al. (56)	2023	SR	Singapore	C, CC and CS	18	0	AD	There is an association between periodontitis and AD.	There is an association between periodontitis and AD; however, causality and the specific roles of either cannot yet be determined.
Said–Sadier et al. (59)	2023	SR	United Arab Emirates	C, CC and CS	11	0	Cognitive impairment, dementia and AD	The results of included studies show that chronic periodontitis patients with at least eight years of exposure are at higher risk of developing cognitive decline, dementia and AD.	All the included studies show evidence of an association between periodontitis and cognitive impairment or dementia and AD.
Larvin et al. (61)	2023	SR and MA	United Kingdom	C and CS	49	49	Cognitive decline	PRR = 1.43 (1.16–1.77)	The findings of this systematic review reveal that PD is associated with cognitive decline, dementia and AD.
								RR = 1.33 (1.13–1.55)	
							Dementia and AD	PRR = 1.08 (0.89–1.31)	
								RR = 1.23 (1.15–1.32)	
							Cognitive decline—mild PD	RR = 0.94 (0.86–1.03)	
							Cognitive decline—moderate PD	PRR = 1.21 (0.85–1.72)	
								RR = 1.14 (1.07–1.22)	
							Cognitive decline—severe PD	PRR = 1.35 (1.07–1.71)	
								RR = 1.25 (1.18–1.32)	
							Cognitive decline—Asia	PRR = 1.25 (0.94–1.67)	
								RR = 1.18 (1.11–1.26)	
							Cognitive decline—Europe	PRR = 1.47 (1.08–2.01)	
								RR = 1.73 (1.22–2.47)	
							Cognitive decline—South America	PRR = 1.85 (0.71–4.84)	
							Cognitive decline—Young	PRR = 1.17 (0.97–1.42)	
								RR = 1.40 (1.01–1.94)	
							Cognitive decline—Older	PRR = 2.06 (1.41–3.02)	
								RR = 1.36 (1.08–1.71)	
							Dementia and AD—mild PD	RR = 1.20 (0.76–1.90)	
							Dementia and AD—moderate PD	PRR = 0.92 (0.69–1.23)	
								RR = 1.13 (1.03–1.23)	
							Dementia and AD—severe PD	PRR = 2.16 (0.72–6.49)	
								RR = 1.13 (1.11–1.19)	
							Dementia and AD—Young	PRR = 1.18 (1.17–1.19)	
								RR = 1.29 (1.16–1.44)	
							Dementia and AD—Older	PRR = 0.99 (0.63–1.55)	
								RR = 1.17 (1.01–1.35)	
							Dementia and AD—Asia	RR = 1.30 (1.16–1.45)	
							Dementia and AD—Europe	RR = 1.41 (0.89–2.22)	
							Dementia and AD—North America	RR = 1.14 (0.98–1.33)	
Li et al. (66)	2023	SR and MA	China	C	21	21	Dementia	RR = 1.15 (1.10–1.20)	This meta-analysis suggests that tooth loss is associated with increased risk of cognitive decline and dementia, as well as the two most common types of dementia and AD.
							Dementia—Asia	RR = 1.15 (1.10–1.21)	
							Dementia—Europe	RR = 1.06 (0.81–1.38)	
							Dementia—North America	RR = 1.01 (0.92–1.12)	
							Dementia—Men	RR = 1.19 (1.09–1.30)	
							Dementia—Women	RR = 1.14 (1.01–1.29)	
							Cognitive decline	RR = 1.20 (1.14–1.26)	
							Cognitive decline—Asia	RR = 1.26 (1.04–1.53)	
							Cognitive decline—Europe	RR = 1.08 (0.93–1.27)	
							Cognitive decline—North America	RR = 1.12 (1.05–1.19)	
							Cognitive decline—Men	RR = 1.10 (1.01–1.18)	
							AD	RR = 1.11 (1.03–1.21)	
							AD—Asia	RR = 1.11 (1.02–1.20)	
							AD—Europe	RR = 1.91 (0.70–5.23)	
							AD—North America	RR = 1.10 (0.47–2.58)	
							AD—Men	RR = 1.28 (1.11–1.49)	
							AD—Women	RR = 1.19 (1.12–1.27)	
Dziedzic et al. (54)	2022	SR and MA	Poland	C, CC and CS	17	7	Cognitive impairment	OR = 1.36 (1.03–1.79)	There is weak evidence of a positive association between periodontitis and an increased risk of dementia or AD.
							AD	OR = 1.03 (0.98–1.07)	
							Dementia	OR = 1.39 (1.02–1.88)	
Kaliamoorthy et al. (52)	2022	SR and MA	India	C and CC	6	3	AD	OR = 1.67 (1.21–2.32)	The results of this review showed a significant association between periodontitis and AD.
Borsa et al. (8)	2021	SR	France	C, CC and CS	5	0	AD	The presence of periodontitis at baseline was associated with AD.	The current review suggests an association between PD and AD.
Hu et al. (39)	2021	SR and MA	China	C, CC and CS	13	13	AD	OR = 1.78 (1.15–2.76)	This meta-analysis indicated that PD was related to an elevated risk of AD and cognitive impairment.
							AD—Mild/Moderate PD	OR = 1.83 (0.93–3.60)	
							AD—Severe PD	OR = 4.89 (1.60–14.97)	
							Mild cognitive impairment	OR = 1.60 (1.24–2.06)	
							Mild cognitive impairment—Mild/Moderate PD	OR = 1.30 (0.94–1.79)	
							Mild cognitive impairment—Severe PD	OR = 2.32 (1.24–4.36)	
Qiu et al. (67)	2020	SR and MA	China	C, CC and CS	11	9	AD	RR = 1.22 (1.13–1.33)	The results of meta-analysis showed that patients with periodontitis had a higer risk of AD.
							AD—Moderate PD	RR = 1.19 (0.98–1.44)	
							AD—Severe PD	RR = 1.54 (1.05–2.26)	
Dioguardi et al. (36)	2019	SR and MA	Italy	CC	9	9	AD	RR = 2.26 (1.70–3.01)	The patients su ering from Alzheimer's disease are characterized by a greater number of lost dental elements and general edentulism.
Leira et al. (58)	2017	SR and MA	Spain	C, CC and CS	5	3	AD	RR = 1.69 (1.21–2.35)	A significant association was observed between PD and AD.
							AD—Moderate PD	RR = 1.86 (0.89–3.91)	
							AD—Severe PD	RR = 2.98 (1.58–5.62)	

SR, systematic review; MA, meta-analysis; CS, cross-sectional; C, cohort; CC, case and control; PD, periodontal disease; AD, Alzheimer disease; OR, odds ratio; RR, risk/rate ratio.

### 3.3 Assessment of methodological quality and quality of evidence

Ten SR (8, 54–56, 58–62, 64) were considered to have high confidence, and six SR (39, 41, 52, 53, 57, 63) had low confidence (Table 4).

**Table 4:** Assessment of the methodological quality and the quality of the evidence of the included systematic reviews.

Authors	Year	AMSTAR-2																Overall confidence
		1	2*	3	4*	5	6	7*	8	9*	10	11*	12	13*	14	15*	16	
Kim et al. (55)	2025	Yes	Yes	Yes	Yes	Yes	No	Yes	Yes	Yes	Yes	Yes	Yes	Yes	Yes	Yes	Yes	High
Melo et al. (57)	2025	Yes	Yes	Yes	Yes	Yes	No	Yes	Yes	Yes	Yes	No meta-anaysis	Yes	Yes	No meta-anaysis	Yes	High	
Fu et al. (65)	2024	Yes	No	Yes	Yes partial	Yes	Yes	Yes	Yes	Yes	Yes	Yes	Yes	Yes	Yes	Yes	Yes	Low
Bouziane et al. (53)	2023	Yes	Yes	Yes	Yes partial	Yes	Yes	Yes	Yes	Yes	Yes	No meta-analysis	Yes	Yes	No meta-analysis	Yes	High	
Ab Malik et al. (60)	2023	Yes	No	Yes	Yes partial	No	No	Yes	Yes	Yes	Yes	No meta-analysis	Yes	Yes	No meta-analysis	Yes	Low	
Nascimento et al. (56)	2023	Yes	Yes	Yes	Yes	Yes	Yes	Yes	Yes	Yes	Yes	No meta-analysis	Yes	Yes	No meta-analysis	Yes	High	
Said–Sadier et al. (59)	2023	Yes	Yes	Yes	Yes partial	Yes	Yes	Yes	Yes	Yes	Yes	No meta-analysis	Yes	Yes	No meta-analysis	Yes	High	
Larvin et al. (61)	2023	Yes	Yes	Yes	Yes partial	Yes	Yes	Yes	Yes	Yes	Yes	Yes	Yes	Yes	Yes	Yes	Yes	High
Li et al. (66)	2023	Yes	No	Yes	Yes partial	Yes	Yes	Yes	Yes	Yes	Yes	Yes	Yes	Yes	Yes	Yes	Yes	Low
Dziedzic et al. (54)	2022	Yes	No	Yes	Yes	Yes	No	Yes	Yes	Yes	Yes	Yes	Yes	Yes	Yes	Yes	Yes	Low
Kaliamoorthy et al. (52)	2022	Yes	Yes	Yes	Yes	Yes	Yes	Yes	Yes	Yes	Yes	Yes	Yes	Yes	Yes	Yes	Yes	High
Borsa et al. (8)	2021	Yes	Yes	Yes	Yes partial	Yes	Yes	Yes	Yes	Yes partial	Yes	No meta-analysis	Yes	Yes	No meta-analysis	Yes	High	
Hu et al. (39)	2021	Yes	No	Yes	Yes	Yes	Yes	Yes	Yes	Yes	Yes	Yes	Yes	Yes	Yes	Yes	Yes	Low
Qiu et al. (67)	2020	Yes	Yes	Yes	Yes partial	Yes	Yes	Yes	Yes	Yes	Yes	Yes	Yes	Yes	Yes	Yes	Yes	High
Dioguardi et al. (36)	2019	Yes	No	Yes	Yes partial	Yes	Yes	Yes	Yes	Yes	Yes	Yes	Yes	Yes	Yes	Yes	Yes	Low
Leira et al. (58)	2017	Yes	Yes	Yes	Yes	Yes	Yes	Yes	Yes	Yes	Yes	Yes	Yes	Yes	Yes	Yes	Yes	High

AMSTAR, A MeaSurement Tool to Assess Systemic Reviews; 1, Did the research questions and inclusion criteria for the review include the components of PICO? 2, Did the report of the review contain an explicit statement that the review methods were established prior to the conduct of the review and did the report justify any significant deviations from the protocol? 3, Did the review authors explain their selection of the study designs for inclusion in the review? 4, Did the review authors use a comprehensive literature search strategy? 5, Did the review authors perform study selection in duplicate? 6, Did the review authors perform data extraction in duplicate? 7, Did the review authors provide a list of excluded studies and justify the exclusions? 8, Did the review authors describe the included studies in adequate detail? 9, Did the review authors use a satisfactory technique for assessing the risk of bias (RoB) in individual studies that were included in the review? 10, Did the review authors report on the sources of funding for the studies included in the review? 11, If meta-analysis was performed, did the review authors use appropriate methods for statistical combination of results? 12, If meta-analysis was performed, did the review authors assess the potential impact of RoB in individual studies on the results of the meta-analysis or other evidence synthesis? 13, Did the review authors account for RoB in primary studies when interpreting/discussing the results of the review? 14, Did the review authors provide a satisfactory explanation for, and discussion of, any heterogeneity observed in the results of the review? 15, If they performed quantitative synthesis, did the review authors carry out an adequate investigation of publication bias (small study bias) and discuss its likely impact on the results of the review? 16, Did the review authors report any potential sources of conflict of interest, including any funding they received for conducting the review? *, critical domain.

### 3.4 Overlapping

A total of 232 primary studies were identified across the included SR. Of these, approximately 75.86% exhibited overlap, having been included in more than one SR. Specifically, 28 studies were duplicated in two reviews, 11 appeared in three, and 6 were present in four. Additionally, 5 studies were included in five reviews, 1 in six, 2 in seven, 1 in eight, and 1 in ten reviews. Detailed information regarding the extent of overlap and the characteristics of the primary studies is provided in Supplementary Appendix A.

### 3.5 Synthesis of results

The summaries of the findings are displayed in Table 5.

**Table 5:** Synthesis of the results of the included studies.

Authors	Outcome	Condition		Association
Kim et al. (55)	AD	OR = 2.17 (1.22–3.87)	PD	Yes
	AD—Severe PD	OR = 6.87 (2.55–18.54)		Yes
	AD—Moderate PD	OR = 0.73 (0.37–1.44)		No
Melo et al. (57)	AD	Four studies supported connection between AD and periodontal inflammatory parameters, one study found no plausible association.	PD	Yes
Fu et al. (65)	AD	OR = 3.89 (0.68–22.31)	Tooth loss	No
Bouziane et al. (53)	AD	All studies except one showed a significant association between PD and the risk of AD onset and progression.	PD	Yes
Ab Malik et al. (60)	Dementia and AD	A higher prevalence of subjects with severe periodontal disease was observed in individuals diagnosed with dementia/AD.	PD	Yes
Nascimento et al. (56)	AD	There is an association between periodontitis and AD.	PD	Yes
Said-Sadier et al. (59)	Cognitive impairment, dementia and AD	The results of included studies show that chronic periodontitis patients with at least eight years of exposure are at higher risk of developing cognitive decline and dementia.	PD	Yes
Larvin et al. (61)	Dementia and AD	RR = 1.23 (1.15–1.32)	PD	Yes
	Dementia and AD—mild PD	RR = 1.20 (0.76–1.90)		No
	Dementia and AD—moderate PD	RR = 1.13 (1.03–1.23)		Yes
	Dementia and AD—severe PD	RR = 1.13 (1.11–1.19)		Yes
	Dementia and AD—Young	RR = 1.29 (1.16–1.44)		Yes
	Dementia and AD—Older	RR = 1.17 (1.01–1.35)		Yes
	Dementia and AD—Asia	RR = 1.30 (1.16–1.45)		Yes
	Dementia and AD—Europe	RR = 1.41 (0.89–2.22)		No
	Dementia and AD—North America	RR = 1.14 (0.98–1.33)		No
Li et al. (66)	AD	RR = 1.11 (1.03–1.21)	Tooth loss	Yes
	AD—Asia	RR = 1.11 (1.02–1.20)		Yes
	AD—Europe	RR = 1.91 (0.70–5.23)		No
	AD—North America	RR = 1.10 (0.47–2.58)		No
	AD—Men	RR = 1.28 (1.11–1.49)		Yes
	AD—Women	RR = 1.19 (1.12–1.27)		Yes
Dziedzic et al. (54)	AD	OR = 1.03 (0.98–1.07)	Tooth loss	No
Kaliamoorthy et al. (52)	AD	OR = 1.67 (1.21–2.32)	PD	Yes
Borsa et al. (8)	AD	The presence of periodontitis at baseline was associated with AD.	PD	Yes
Hu et al. (39)	AD	OR = 1.78 (1.15–2.76)	PD	Yes
	AD—Mild/Moderate PD	OR = 1.83 (0.93–3.60)		No
	AD—Severe PD	OR = 4.89 (1.60–14.97)		Yes
Qiu et al. (67)	AD	RR = 1.22 (1.13–1.33)	PD	Yes
	AD—Moderate PD	RR = 1.19 (0.98–1.44)		No
	AD—Severe PD	RR = 1.54 (1.05–2.26)		Yes
Dioguardi et al. (36)	AD	RR = 2.26 (1.70–3.01)	Tooth loss	Yes
Leira et al. (58)	AD	RR = 1.69 (1.21–2.35)	PD	Yes
	AD—Moderate PD	RR = 1.86 (0.89–3.91)		No
	AD—Severe PD	RR = 2.98 (1.58–5.62)		Yes

PD, periodontal disease; AD, Alzheimer disease; OR, odds ratio; RR, risk/rate ratio.

### 3.6 Alzheimer disease (AD)

Nine SR (8, 41, 54–56, 58–61) included reported that there was an association between PD and AD, but in 1 SR (54) this association was not found. Six SR (41, 54, 55, 57, 58, 61) meta-analyzed the results and found that the OR ranged from 1.03 [CI: 0.98–1.07] (57) to 2.17 [CI: 1.22–3.87] (58) and the RR ranged from 1.22 [CI: 1.13–1.33] (54) to 1.69 [CI: 1.21–2.35] (61). Melo et al. (60), Bouziane et al. (56), Nascimento et al. (59) and Borsa et al. (8) reported that there is an association between AD and PD.

One SR (63) included reported that there was an association between PD and AD for Asia, but not Europe and North America. This study meta-analyzed its results and found that the RR for Asia was 1.11 [CI: 1.02–1.20], for Europe was 1.91 [CI: 0.70–5.23], and for North America was 1.10 [CI: 0.47–2.58].

One SR (53) included reported that there was an association between PD and AD for sex. This studies meta-analyzed its results and found that the RR for men was 1.28 [CI: 1.11–1.49] and for women was 1.19 [CI: 1.12–1.27].

Four SR (41, 54, 58, 61) included reported that there was an association between PD and AD for severe PD, but not mild or moderate PD. This studies meta-analyzed its results and found that the OR for mild—moderate PD ranged from 0.73 [CI: 0.37–1.44] (58) to 1.83 [CI: 0.93–3.60] (41) and the RR ranged from 1.19 [CI: 0.98–1.44] (54) to 1.86 [CI: 0.89–3.91] (61), and for severe PD the OR ranged from 4.89 [CI: 1.60–14.97] (41) to 6.87 [CI: 2.55–18.54] (58) and the RR ranged from 1.54 [CI: 1.05–2.26] (54) to 2.98 [CI: 1.58–5.62] (61).

Two SR (38, 53) included reported that there was an association between tooth loss and AD, but in 1 SR (52) this association was not found. These studies meta-analyzed their results and found that the OR was 3.89 [CI: 0.68–22.31] (52) and the RR ranged from 1.11 [CI: 1.03–1.21] (53) to 2.26 [CI: 1.70–3.01] (38).

### 3.7 Dementia and Ad

Three SR (62–64) included reported that there was an association between PD and cognitive impairment, dementia and AD. One SR (64) meta-analyzed its results and found that the RR was 1.23 [CI: 1.15–1.32]. Ab Malik et al. (63) reported that there is an association between PD and dementia and AD, and Said-Sadier et al. (62) reported that there is an association between PD and cognitive impairment, dementia and AD.

One SR (64) included reported that there was an association between PD and dementia and AD for Asia, but not Europe and North America. This study meta-analyzed its results and found that the RR for Asia was 1.30 [CI: 1.16–1.45], for Europe was 1.41 [CI: 0.89–2.22], and for North America was 1.14 [CI: 0.98–1.33].

One SR (64) included reported that there was an association between PD and dementia and AD for age. These studies meta-analyzed its results and found that the RR for young was 1.29 [CI: 1.16–1.44] and for older was 1.17 [CI: 1.01–1.35].

One SR (64) included reported that there was an association between PD and dementia and AD for moderate or severe PD, but not mild PD. This study meta-analyzed its results and found that the RR for mild PD was 1.20 [CI: 0.76–1.90], for moderate PD was 1.13 [CI: 1.03–1.23], and for severe PD was 1.13 [CI: 1.11–1.19].

## 4 Discussion

This umbrella review aimed to elucidate the link between PD and AD by gathering and examining pertinent systematic reviews and meta-analysis. From this investigation, we distilled these principal findings: the systematic reviews included here corroborate an association between PD and AD, aligning with the evidence presented by Hernández-Nieto et al. (14).

The investigation involved an exhaustive literature search aimed at gathering and critically evaluating all existing systematic reviews on the relationship between PD and AD. Sixteen SRs were identified that met the established selection criteria. Although SRs constitute a reliable source of scientific evidence, it is important to exercise caution when interpreting their results due to the possibility of bias (68). The SRs considered in this research were subject to certain limitations stemming from the chosen primary studies-for instance, the inclusion of various types of studies, the fact that some of them did not present AD as a primary outcome but addressed it in conjunction with dementia and cognitive impairment, and the high overlap of primary studies. These limitations within the primary studies impeded the feasibility of performing a meta-analysis. Although several of the reviews analysed demonstrated high confidence ratings-potentially bolstering the study's findings and conclusions-the fact that numerous systematic reviews exhibited low confidence underscores the imperative for more stringent research methods in this field. We evaluated the methodological rigor of the included SRs with the updated and well-established AMSTRAR-2 instrument (69). Weaknesses were identified in critical domain 2, which included the lack of an explicit statement on the establishment of review methods prior to the review. In addition, deficiencies were found in non-critical domains 5 and 6, which included the lack of selection and data extraction from duplicate studies. These results emphasize the necessity of resolving these methodological challenges in upcoming systematic reviews (SRs).

An umbrella review conducted in 2024 (14), which included 13 systematic reviews on the association between PD and AD, highlighted that, although there is a relationship between AD and PD according to the studies analyzed, more support is needed in the literature and the inclusion of randomized clinical trials on this topic.

Interest in exploring the link between PD and AD. Has surged in recent years, with extensive research confirming this connection. AD now stands as the leading cause of dementia and ranks among the top causes of death in older adults globally, impacting individuals across all racial and ethnic groups (1, 8). While prevention remains elusive and prevalence rises with age, evidence indicates that modifiable risk factors can be addressed and cognitive health supported (70, 71).

Dental health researchers have long investigated the link between AD and PD, identifying two main mechanistic pathways: one hand, the gradual loss of cognitive function in patients may lead to poorer oral hygiene practices and thus elevate periodontal risk; on the other hand, chronic immune-driven inflammation originating in periodontitis can trigger neuroinflammatory responses that promote Alzheimer's pathology (14).

Care must be taken when interpreting the results of SRs, as approximately 76% of the primary studies are recurrently included in multiple reviews. This redundancy may lead to repeated analyses of the same data, which could bias the perceived scope and depth of research activity in this field. Nevertheless, the development of new SRs remains warranted, particularly those designed to address the methodological limitations noted by Moher (72), given the considerable degree of overlap identified among existing reviews.

As individuals age, the link between PD and AD becomes more pronounced. The likely cause is that aging gradually impairs the immune system and the body's protective barriers. This facilitates the persistence of chronic infections such as PD and, simultaneously, the accumulation of neuroinflammatory damage. Furthermore, with age, the cognitive and physical abilities to maintain adequate oral hygiene decline, which aggravates PD and, consequently, enhances systemic inflammation that can affect the brain (73–75).

Furthermore, this association was found to be common in both men and women. This could be explained by the universality of shared risk factors, such as advanced age, chronic systemic inflammation, and altered immune response. Furthermore, periodontal bacteria and their neuroinflammatory effects do not discriminate by gender, with both men and women having similar exposures to oral pathogens and systemic risk factors related to AD (57, 76).

Additionally, this association was observed to be common in people suffering from severe PD. This could be explained by the fact that in severe cases of PD, damage to periodontal tissues allows for greater release of bacteria and toxins into the bloodstream, which intensifies systemic inflammation. This persistent inflammation can reach the central nervous system and trigger or aggravate neurodegenerative processes related to AD. Studies have shown that the presence of bacteria such as *P. gingivalis* in the brain is related to more severe cases of cognitive decline (39, 40, 77).

Finally, the association was observed to be present in Asia. This could be attributed to cultural, dietary, and genetic factors that influence both oral health and the predisposition to AD. High rates of population aging in several Asian countries, along with reduced access to preventive dental care in some regions, may also play a role, favoring severe forms of PD and, consequently, a higher likelihood of AD-associated neuroinflammation (78, 79).

### 4.1 Implications for clinical practice

Patients with Alzheimeŕs disease present unique challenges to oral care providers. These includes cognitive decline leading to poor cooperation during procedures, difficulties in understanding and following hygiene instructions, increase risk of aspiration during treatments, and the need for caregiver involvement in daily oral care routines. Behavioral management techniques, shorter appointment times, and frequent preventive visits are often required.

Oral health practitioners are tasked with informing and empowering patients about the link between PD and AD. Encouraging consistent oral hygiene-brushing, flossing, and using mouthwash- helps control plaque formation and may lower Alzheimer's risk. In personalized care, it's advisable to include periodontal evaluations in routine risk screening alongside patient education on Alzheimer's. implementing preventive actions to modify risk factors is essential to reduce the chances of both conditions. Additionally, creating a follow-up protocol for Alzheimer's patients—including regular dental exams and timely periodontal assessments-is key. Working in tandem with psychiatrists, nutritionists, and other health professionals ensures a comprehensive, coordinated approach to the dental and medical management of Alzheimer's patients.

### 4.2 Implications for research

This review highlights the critical need to enhance the quality of SR reporting. To address this need, the authors advocate the adoption of robust quality assessment tools to guide the development of future SRs. They stress that primary studies should be conducted with rigorous methodological standards to ensure reliable results.

For future research in this field, the authors recommend conducting high-quality prospective studies with large sizes and standardized measurements. They also call for more comprehensive investigations to clarify the precise mechanisms and the scope of the relationship between PD and AD.

### 4.3 Limitations

This umbrella review has some limitations. First, many of the included systematic reviews were based on observational studies, which, despite revealing associations, do not confirm causality. Second, there was high overlap among primary studies (over 75%), potentially inflating the evidence base and reducing independence across reviews. Third, meta-analyses were not available for all included systematic reviews, and where present, they often varied in effect measures and population characteristics. Fourth, diagnostic criteria for both PD and AD differed across studies, contributing to clinical heterogeneity and limiting comparability. Finally, some reviews lacked protocol registration or risk of bias assessment, which reduces confidence in their findings. Future systematic reviews should follow standardized protocols (e.g., PRISMA, AMSTAR 2) to improve methodological rigor.

Despite these limitations, this umbrella review provides a structures synthesis of the best available evidence, guiding future research and informing clinical awareness.

## 5 Conclusion

Drawing from the findings and conclusions of the including systematic reviews-many of which demonstrated high methodological confidence- there is consistent observational evidence supporting an association between periodontal disease and Alzheimer's disease. This relationship appears to be bidirectional, as cognitive impairment affects oral hygiene practices, while chronic periodontal inflammation may contribute to neurodegeneration. Given the aging global population and the growing burden of Alzheimeŕs disease, early periodontal evaluation and management should be considered part of comprehensive geriatric care. Additionally, interdisciplinary strategies involving dental professionals, neurologists, caregivers, and primary healthcare providers are essential for improving the quality of life and clinical outcomes of these patients. Future research should aim to clarify causal mechanisms and evaluate the effectiveness of preventive periodontal interventions in reducing Alzheimeŕs disease progression.

## Data Availability

The original contributions presented in the study are included in the article/Supplementary Material, further inquiries can be directed to the corresponding author.
