# Supplementary material for: Association between periodontal disease and Alzheimer's disease: umbrella review
Source: Front Dent Med. 2025 Jul 9;6:1635200. doi: 10.3389/fdmed.2025.1635200 (PMC12283705; doi:10.3389/fdmed.2025.1635200)
Supplement: Supplementary file 1 [file Table1.docx]

**Appendix A.** Overlapping of primary studies in systematic reviews.

| **Primary Studies** | **Systematic Reviews That Included the Primary Studies** | **Times That Primary Studies Were Included** |
| --- | --- | --- |
| Gil–Montoya et al. (1) | Kim et al. (2), Bouziane et al. (3), Ab Malik et al. (4), Nascimento et al. (5), Dziedzic et al. (6), Kaliamoorthy et al. (7), Hu et al. (8), Qiu et al. (9), Dioguardi et al. (10), Leira et al. (11) | 10 |
| de Souza et al. (12) | Kim et al. (2), Ab Malik et al. (4), Nascimento et al. (5), Kaliamoorthy et al. (7), Hu et al. (8), Qiu et al. (9), Dioguardi et al. (10), Leira et al. (11) | 8 |
| Chen et al. (13) | Kim et al. (2), Bouziane et al. (3), Said–Sadier et al. (14), Larvin et al. (15), Dziedzic et al. (6), Hu et al. (8), Qiu et al. (9) | 7 |
| Holmer et al. (16) | Kim et al. (2), Bouziane et al. (3), Ab Malik et al. (4), Nascimento et al. (5), Dziedzic et al. (6), Hu et al. (8), Qiu et al. (9) | 7 |
| Syrjälä et al. (17) | Melo et al. (18), Nascimento et al. (5), Kaliamoorthy et al. (7), Borsa et al. (19), Qiu et al. (9), Leira et al. (11) | 6 |
| Arrivé et al. (20) | Kim et al. (2), Bouziane et al. (3), Larvin et al. (15), Li et al. (21), Dziedzic et al. (6) | 5 |
| Tzeng et al. (22) | Kim et al. (2), Bouziane et al. (3), Said–Sadier et al. (14), Larvin et al. (15), Dziedzic et al. (6) | 5 |
| Aragón et al. (23) | Kim et al. (2), Ab Malik et al. (4), Nascimento et al. (5), Qiu et al. (9), Dioguardi et al. (10) | 5 |
| Martande et al. (24) | Melo et al. (18), Nascimento et al. (5), Kaliamoorthy et al. (7), Qiu et al. (9), Leira et al. (11) | 5 |
| Choi et al. (25) | Kim et al. (2), Said–Sadier et al. (14), Larvin et al. (15), Dziedzic et al. (6), Hu et al. (8) | 5 |
| Ide et al. (26) | Kim et al. (2), Bouziane et al. (3), Kaliamoorthy et al. (7), Borsa et al. (19) | 4 |
| Cestari et al. (27) | Kim et al. (2), Ab Malik et al. (4), Nascimento et al. (5), Hu et al. (8) | 4 |
| Stewart et al. (28) | Kim et al. (2), Said–Sadier et al. (14), Li et al. (21), Dziedzic et al. (6) | 4 |
| Stein et al. (29) | Fu et al. (30), Larvin et al. (15), Li et al. (21), Dziedzic et al. (6) | 4 |
| Iwasaki et al. (31) | Kim et al. (2), Larvin et al. (15), Dziedzic et al. (6), Hu et al. (8) | 4 |
| Nilsson et al. (32) | Fu et al. (30), Larvin et al. (15), Dziedzic et al. (6), Hu et al. (8) | 4 |
| Lopez–Jornet et al. (33) | Kim et al. (2), Fu et al. (30), Ab Malik et al. (4), | 3 |
| Chu et al. (34) | Kim et al. (2), Ab Malik et al. (4), Larvin et al. (15) | 3 |
| de Oliveira et al. (35) | Kim et al. (2), Nascimento et al. (5), Hu et al. (8) | 3 |
| Panzarella et al. (36) | Kim et al. (2), Nascimento et al. (5), Borsa et al. (19) | 3 |
| Shin et al. (37) | Said–Sadier et al. (14), Larvin et al. (15), Hu et al. (8) | 3 |
| Okamoto et al. (38) | Fu et al. (30), Larvin et al. (15), Hu et al. (8) | 3 |
| Saito et al. (39) | Fu et al. (30), Larvin et al. (15), Li et al. (21) | 3 |
| Yoo et al. (40) | Fu et al. (30), Larvin et al. (15), Li et al. (21) | 3 |
| Lee et al. (41) | Kim et al. (2), Larvin et al. (15), Dziedzic et al. (6) | 3 |
| Lee et al. (42) | Kim et al. (2), Larvin et al. (15), Dziedzic et al. (6) | 3 |
| Ship et al. (43) | Nascimento et al. (5), Qiu et al. (9), Leira et al. (11) | 3 |
| Marruganti et al. (44) | Melo et al. (18), Fu et al. (30) | 2 |
| Bramanti et al. (45) | Kim et al. (2), Ab Malik et al. (4) | 2 |
| Ship (46) | Ab Malik et al. (4), Nascimento et al. (5) | 2 |
| D'Alessandro et al. (47) | Kim et al. (2), Nascimento et al. (5) | 2 |
| Barbe et al. (48) | Kim et al. (2), Larvin et al. (15) | 2 |
| Nilsson et al. (49) | Kim et al. (2), Larvin et al. (15) | 2 |
| Demmer et al. (50) | Said–Sadier et al. (14), Larvin et al. (15) | 2 |
| Tsuneishi et al. (51) | Fu et al. (30), Larvin et al. (15) | 2 |
| Nilsson et al. (52) | Fu et al. (30), Larvin et al. (15) | 2 |
| Hatta et al. (53) | Fu et al. (30), Larvin et al. (15) | 2 |
| Xu et al. (54) | Fu et al. (30), Larvin et al. (15) | 2 |
| Kim et al. (55) | Larvin et al. (15), Li et al. (21) | 2 |
| Paganini–Hill et al. (56) | Larvin et al. (15), Li et al. (21) | 2 |
| Yamamoto et al. (57) | Larvin et al. (15), Li et al. (21) | 2 |
| Batty et al. (58) | Larvin et al. (15), Li et al. (21) | 2 |
| Stewart et al. (59) | Larvin et al. (15), Li et al. (21) | 2 |
| Takeuchi et al. (60) | Larvin et al. (15), Li et al. (21) | 2 |
| Kim et al. (61) | Larvin et al. (15), Li et al. (21) | 2 |
| Kiuchi et al. (62) | Larvin et al. (15), Li et al. (21) | 2 |
| Yang et al. (63) | Larvin et al. (15), Li et al. (21) | 2 |
| Lee et al. (64) | Larvin et al. (15), Dziedzic et al. (6) | 2 |
| Okamoto et al. (65) | Larvin et al. (15), Dziedzic et al. (6) | 2 |
| Iwasaki et al. (66) | Fu et al. (30), Dziedzic et al. (6) | 2 |
| Kaye et al. (67) | Li et al. (21), Dziedzic et al. (6) | 2 |
| Sparks Stein et al. (68) | Said–Sadier et al. (14), Kaliamoorthy et al. (7) | 2 |
| ALFotawi et al. (69) | Larvin et al. (15), Hu et al. (8) | 2 |
| Tiisanoja et al. (70) | Larvin et al. (15), Hu et al. (8) | 2 |
| Rai et al. (71) | Ab Malik et al. (4), Dioguardi et al. (10) | 2 |

References

1. Gil-Montoya JA, Sanchez-Lara I, Carnero-Pardo C, Fornieles F, Montes J, Vilchez R, et al. Is periodontitis a risk factor for cognitive impairment and dementia? A case-control study. J Periodontol 2015;86:244–53. doi: 10.1902/jop.2014.140340.

2. Kim DONG-HEE, Han GYEO. Periodontitis as a risk factor for dementia: A systematic review and meta-analysis. J Evid-Based Dent Pract 2025;25. doi: 10.1016/j.jebdp.2025.102094.

3. Bouziane A, Lattaf S, Abdallaoui Maan L. Effect of Periodontal Disease on Alzheimer’s Disease: A Systematic Review. Cureus 2023;15:e46311. doi: 10.7759/cureus.46311.

4. Ab Malik N, Walls AWG. Periodontal health status of people with dementia - A systematic review of case-control studies. Saudi Dent J 2023;35:625–40. doi: 10.1016/j.sdentj.2023.06.004.

5. Nascimento GG, Leite FRM, Mesquita CM, Vidigal MTC, Borges GH, Paranhos LR. Confounding in observational studies evaluating the association between Alzheimer’s disease and periodontal disease: A systematic review. Heliyon 2023;9:e15402. doi: 10.1016/j.heliyon. 2023.e15402.

6. Dziedzic A. Is Periodontitis Associated with Age-Related Cognitive Impairment? The Systematic Review, Confounders Assessment and Meta-Analysis of Clinical Studies. Int J Mol Sci 2022;23:15320. doi: 10.3390/ijms232315320.

7. Kaliamoorthy S, Nagarajan M, Sethuraman V, Jayavel K, Lakshmanan V, Palla S. Association of Alzheimer’s disease and periodontitis - a systematic review and meta-analysis of evidence from observational studies. Med Pharm Rep 2022;95:144–51. doi: 10.15386/mpr-2278.

8. Hu X, Zhang J, Qiu Y, Liu Z. Periodontal disease and the risk of Alzheimer’s disease and mild cognitive impairment: a systematic review and meta-analysis. Psychogeriatrics 2021;21:813–25. doi: 10.1111/psyg.12743.

9. Qiu C, Zhou W, Shi W-T, Song Z-C. Association between periodontitis and Alzheimer disease: a meta analysis. Shanghai Kou Qiang Yi Xue Shanghai J Stomatol 2020;29:661–8. doi:10.19439/j.sjos.2020.06.020.

10. Dioguardi M, Gioia GD, Caloro GA, Capocasale G, Zhurakivska K, Troiano G, et al. The Association between Tooth Loss and Alzheimer’s Disease: a Systematic Review with Meta-Analysis of Case Control Studies. Dent J 2019;7:49. doi: 10.3390/dj7020049.

11. Leira Y, Domínguez C, Seoane J, Seoane-Romero J, Pías-Peleteiro JM, Takkouche B, et al. Is Periodontal Disease Associated with Alzheimer’s Disease? A Systematic Review with Meta-Analysis. Neuroepidemiology 2017;48:21–31. doi: 10.1159/000458411.

12. de Souza Rolim T, Fabri GMC, Nitrini R, Anghinah R, Teixeira MJ, de Siqueira JTT, et al. Oral infections and orofacial pain in Alzheimer’s disease: a case-control study. J Alzheimers Dis JAD 2014;38:823–9. doi: 10.3233/JAD-131283.

13. Chen C-K, Wu Y-T, Chang Y-C. Association between chronic periodontitis and the risk of Alzheimer’s disease: a retrospective, population-based, matched-cohort study. Alzheimers Res Ther 2017;9:56. doi: 10.1186/s13195-017-0282-6.

14. Said-Sadier N, Sayegh B, Farah R, Abbas LA, Dweik R, Tang N, et al. Association between Periodontal Disease and Cognitive Impairment in Adults. Int J Environ Res Public Health 2023;20:4707. doi: 10.3390/ijerph20064707.

15. Larvin H, Gao C, Kang J, Aggarwal VR, Pavitt S, Wu J. The impact of study factors in the association of periodontal disease and cognitive disorders: systematic review and meta-analysis. Age Ageing 2023;52:afad015. doi: 10.1093/ageing/afad015.

16. Holmer J, Eriksdotter M, Schultzberg M, Pussinen PJ, Buhlin K. Association between periodontitis and risk of Alzheimer’s disease, mild cognitive impairment and subjective cognitive decline: A case-control study. J Clin Periodontol 2018;45:1287–98. doi: 10.1111/jcpe.13016.

17. Syrjälä A-MH, Ylöstalo P, Ruoppi P, Komulainen K, Hartikainen S, Sulkava R, et al. Dementia and oral health among subjects aged 75 years or older. Gerodontology 2012;29:36–42. doi: 10.1111/j.1741-2358.2010.00396.x.

18. Melo A, Flores-Fraile J, Lo Giudice R, Marchetti E, Nart J, Greethurst Ar, et al. Association Between Alzheimer’s Disease and Periodontal Inflammatory Parameters: A Systematic Review. J Clin Exp Dent 2025:e310–23. doi: 10.4317/jced.62519.

19. Borsa L, Dubois M, Sacco G, Lupi L. Analysis the Link between Periodontal Diseases and Alzheimer’s Disease: A Systematic Review. Int J Environ Res Public Health 2021;18:9312. doi: 10.3390/ijerph18179312.

20. Arrivé E, Letenneur L, Matharan F, Laporte C, Helmer C, Barberger-Gateau P, et al. Oral health condition of French elderly and risk of dementia: a longitudinal cohort study. Community Dent Oral Epidemiol 2012;40:230–8. doi: 10.1111/j.1600-0528.2011.00650.x.

21. Li L, Zhang Q, Yang D, Yang S, Zhao Y, Jiang M, et al. Tooth loss and the risk of cognitive decline and dementia: A meta-analysis of cohort studies. Front Neurol 2023;14. doi: 10.3389/fneur.2023.1103052.

22. Tzeng N-S, Chung C-H, Yeh C-B, Huang R-Y, Yuh D-Y, Huang S-Y, et al. Are Chronic Periodontitis and Gingivitis Associated with Dementia? A Nationwide, Retrospective, Matched-Cohort Study in Taiwan. Neuroepidemiology 2016;47:82–93. doi: 10.1159/000449166.

.23. Aragón F, Zea-Sevilla MA, Montero J, Sancho P, Corral R, Tejedor C, et al. Oral health in Alzheimer’s disease: a multicenter case-control study. Clin Oral Investig 2018;22:3061–70. doi: 10.1007/s00784-018-2396-z.

24. Martande SS, Pradeep AR, Singh SP, Kumari M, Suke DK, Raju AP, et al. Periodontal health condition in patients with Alzheimer’s disease. Am J Alzheimers Dis Other Demen 2014;29:498–502. doi: 10.1177/1533317514549650.

25. Choi S, Kim K, Chang J, Kim SM, Kim SJ, Cho H-J, et al. Association of Chronic Periodontitis on Alzheimer’s Disease or Vascular Dementia. J Am Geriatr Soc 2019;67:1234–9. doi: 10.1111/jgs.15828.

26. Ide M, Harris M, Stevens A, Sussams R, Hopkins V, Culliford D, et al. Periodontitis and Cognitive Decline in Alzheimer’s Disease. PloS One 2016;11:e0151081. doi: 10.1371/journal.pone.0151081.

27. Cestari JAF, Fabri GMC, Kalil J, Nitrini R, Jacob-Filho W, de Siqueira JTT, et al. Oral Infections and Cytokine Levels in Patients with Alzheimer’s Disease and Mild Cognitive Impairment Compared with Controls. J Alzheimers Dis JAD 2016;52:1479–85. doi: 10.3233/JAD-160212.

28. Stewart R, Weyant RJ, Garcia ME, Harris T, Launer LJ, Satterfield S, et al. Adverse oral health and cognitive decline: the health, aging and body composition study. J Am Geriatr Soc 2013;61:177–84. doi: 10.1111/jgs.12094.

29. Stein PS, Desrosiers M, Donegan SJ, Yepes JF, Kryscio RJ. Tooth loss, dementia and neuropathology in the Nun study. J Am Dent Assoc 1939 2007;138:1314–22; quiz 1381–2. doi: 10.14219/jada.archive.2007.0046.

30. Fu Y-D, Li C-L, Hu C-L, Pei M-D, Cai W-Y, Li Y-Q, et al. Meta Analysis of the Correlation between Periodontal Health and Cognitive Impairment in the Older Population. J Prev Alzheimers Dis 2024;11:1307–15. doi: 10.14283/jpad.2024.87.

31. Iwasaki M, Kimura Y, Ogawa H, Yamaga T, Ansai T, Wada T, et al. Periodontitis, periodontal inflammation, and mild cognitive impairment: A 5-year cohort study. J Periodontal Res 2019;54:233–40. doi: 10.1111/jre.12623.

32. Nilsson H, Berglund JS, Renvert S. Periodontitis, tooth loss and cognitive functions among older adults. Clin Oral Investig 2018;22:2103–9. doi: 10.1007/s00784-017-2307-8.

33. Lopez-Jornet P, Zamora Lavella C, Pons-Fuster Lopez E, Tvarijonaviciute A. Oral Health Status in Older People with Dementia: A Case-Control Study. J Clin Med 2021;10:477. doi: 10.3390/jcm10030477.

34. Chu CH, Ng A, Chau AMH, Lo ECM. Oral health status of elderly chinese with dementia in Hong Kong. Oral Health Prev Dent 2015;13:51–7. doi: 10.3290/j.ohpd.a32343.

35. de Oliveira Araújo R, Villoria GEM, Luiz RR, Esteves JC, Leão ATT, Feres-Filho EJ. Association between periodontitis and Alzheimer’s disease and its impact on the self-perceived oral health status: a case-control study. Clin Oral Investig 2021;25:555–62. doi: 10.1007/s00784-020-03489-w.

36. Panzarella V, Mauceri R, Baschi R, Maniscalco L, Campisi G, Monastero R. Oral Health Status in Subjects with Amnestic Mild Cognitive Impairment and Alzheimer’s Disease: Data from the Zabút Aging Project. J Alzheimers Dis JAD 2022;87:173–83. doi: 10.3233/JAD-200385.

37. Shin H-S, Shin M-S, Ahn Y-B, Choi B-Y, Nam J-H, Kim H-D. Periodontitis Is Associated with Cognitive Impairment in Elderly Koreans: Results from the Yangpyeong Cohort Study. J Am Geriatr Soc 2016;64:162–7. doi: 10.1111/jgs.13781.

38. Okamoto N, Morikawa M, Okamoto K, Habu N, Iwamoto J, Tomioka K, et al. Relationship of tooth loss to mild memory impairment and cognitive impairment: findings from the Fujiwara-kyo study. Behav Brain Funct BBF 2010;6:77. doi: 10.1186/1744-9081-6-77.

39. Saito S, Ohi T, Murakami T, Komiyama T, Miyoshi Y, Endo K, et al. Association between tooth loss and cognitive impairment in community-dwelling older Japanese adults: a 4-year prospective cohort study from the Ohasama study. BMC Oral Health 2018;18:142. doi: 10.1186/s12903-018-0602-7.

40. Yoo J-J, Yoon J-H, Kang M-J, Kim M, Oh N. The effect of missing teeth on dementia in older people: a nationwide population-based cohort study in South Korea. BMC Oral Health 2019;19:61. doi: 10.1186/s12903-019-0750-4.

41. Lee Y-L, Hu H-Y, Huang L-Y, Chou P, Chu D. Periodontal Disease Associated with Higher Risk of Dementia: Population-Based Cohort Study in Taiwan. J Am Geriatr Soc 2017;65:1975–80. doi: 10.1111/jgs.14944.

42. Lee Y-T, Lee H-C, Hu C-J, Huang L-K, Chao S-P, Lin C-P, et al. Periodontitis as a Modifiable Risk Factor for Dementia: A Nationwide Population-Based Cohort Study. J Am Geriatr Soc 2017;65:301–5. doi: 10.1111/jgs.14449.

43. Ship JA, Puckett SA. Longitudinal study on oral health in subjects with Alzheimer’s disease. J Am Geriatr Soc 1994;42:57–63. doi: 10.1111/j.1532-5415.1994.tb06074.x.

44. Marruganti C, Baima G, Aimetti M, Grandini S, Sanz M, Romandini M. Periodontitis and low cognitive performance: A population-based study. J Clin Periodontol 2023;50:418–29. doi: 10.1111/jcpe.13779.

45. Bramanti E, Bramanti A, Matacena G, Bramanti P, Rizzi A, Cicciù M. Clinical evaluation of the oral health status in vascular-type dementia patients. A case-control study. Minerva Stomatol 2015;64:167–75.

46. Ship JA. Oral health of patients with Alzheimer’s disease. J Am Dent Assoc 1939 1992;123:53–8. doi: 10.14219/jada.archive.1992.0005.

47. D’Alessandro G, Costi T, Alkhamis N, Bagattoni S, Sadotti A, Piana G. Oral Health Status in Alzheimer’s Disease Patients: A Descriptive Study in an Italian Population. J Contemp Dent Pract 2018;19:483–9.

48. Barbe AG, Küpeli LS, Hamacher S, Noack MJ. Impact of regular professional toothbrushing on oral health, related quality of life, and nutritional and cognitive status in nursing home residents. Int J Dent Hyg 2020;18:238–50. doi: 10.1111/idh.12439.

49. Nilsson H, Sanmartin Berglund J, Renvert S. Longitudinal evaluation of periodontitis and development of cognitive decline among older adults. J Clin Periodontol 2018;45:1142–9. doi: 10.1111/jcpe.12992.

50. Demmer RT, Norby FL, Lakshminarayan K, Walker KA, Pankow JS, Folsom AR, et al. Periodontal disease and incident dementia: The Atherosclerosis Risk in Communities Study (ARIC). Neurology 2020;95:e1660–71. doi: 10.1212/WNL.0000000000010312.

51. Tsuneishi M, Yamamoto T, Yamaguchi T, Kodama T, Sato T. Association between number of teeth and Alzheimer’s disease using the National Database of Health Insurance Claims and Specific Health Checkups of Japan. PloS One 2021;16:e0251056. doi: 10.1371/journal.pone.0251056.

52. Nilsson H, Berglund J, Renvert S. Tooth loss and cognitive functions among older adults. Acta Odontol Scand 2014;72:639–44. doi: 10.3109/00016357.2014.882983.

53. Hatta K, Ikebe K, Gondo Y, Kamide K, Masui Y, Inagaki H, et al. Influence of lack of posterior occlusal support on cognitive decline among 80-year-old Japanese people in a 3-year prospective study. Geriatr Gerontol Int 2018;18:1439–46. doi: 10.1111/ggi.13508.

54. Xu S, Huang X, Gong Y, Sun J. Association between tooth loss rate and risk of mild cognitive impairment in older adults: a population-based longitudinal study. Aging 2021;13:21599–609. doi: 10.18632/aging.203504.

55. Kim DH, Jeong SN, Lee JH. Severe periodontitis with tooth loss as a modifiable risk factor for the development of Alzheimer, vascular, and mixed dementia: National Health Insurance Service-National Health Screening Retrospective Cohort 2002-2015. J Periodontal Implant Sci 2020;50:303–12. doi: 10.5051/jpis.2000600030.

56. Paganini-Hill A, White SC, Atchison KA. Dentition, dental health habits, and dementia: the Leisure World Cohort Study. J Am Geriatr Soc 2012;60:1556–63. doi: 10.1111/j.1532-5415.2012.04064.x.

57. Yamamoto T, Kondo K, Hirai H, Nakade M, Aida J, Hirata Y. Association between self-reported dental health status and onset of dementia: a 4-year prospective cohort study of older Japanese adults from the Aichi Gerontological Evaluation Study (AGES) Project. Psychosom Med 2012;74:241–8. doi: 10.1097/PSY.0b013e318246dffb.

58. Batty G-D, Li Q, Huxley R, Zoungas S, Taylor B-A, Neal B, et al. Oral disease in relation to future risk of dementia and cognitive decline: prospective cohort study based on the Action in Diabetes and Vascular Disease: Preterax and Diamicron Modified-Release Controlled Evaluation (ADVANCE) trial. Eur Psychiatry J Assoc Eur Psychiatr 2013;28:49–52. doi: 10.1016/j.eurpsy.2011.07.005.

59. Stewart R, Stenman U, Hakeberg M, Hägglin C, Gustafson D, Skoog I. Associations between oral health and risk of dementia in a 37-year follow-up study: the prospective population study of women in Gothenburg. J Am Geriatr Soc 2015;63:100–5. doi: 10.1111/jgs.13194.

60. Takeuchi K, Ohara T, Furuta M, Takeshita T, Shibata Y, Hata J, et al. Tooth Loss and Risk of Dementia in the Community: the Hisayama Study. J Am Geriatr Soc 2017;65:e95–100. doi: 10.1111/jgs.14791.

61. Kim JH, Oh JK, Wee JH, Kim YH, Byun S-H, Choi HG. Association between Tooth Loss and Alzheimer’s Disease in a Nested Case-Control Study Based on a National Health Screening Cohort. J Clin Med 2021;10:3763. doi: 10.3390/jcm10173763.

62). Kiuchi S, Cooray U, Kusama T, Yamamoto T, Abbas H, Nakazawa N, et al. Oral Status and Dementia Onset: Mediation of Nutritional and Social Factors. J Dent Res 2022;101:420–7. doi: 10.1177/00220345211049399.

63. Yang H-L, Li F-R, Chen P-L, Cheng X, Mao C, Wu X-B. Tooth Loss, Denture Use, and Cognitive Impairment in Chinese Older Adults: A Community Cohort Study. J Gerontol A Biol Sci Med Sci 2022;77:180–7. doi: 10.1093/gerona/glab056.

64. Lee C-Y, Chang C-C, Lin C-S, Yeh C-C, Hu C-J, Wu C-Z, et al. Risk of dementia in patients with periodontitis and related protective factors: A nationwide retrospective cohort study. J Clin Periodontol 2020;47:1428–36. doi: 10.1111/jcpe.13372.

65. Okamoto N, Morikawa M, Tomioka K, Yanagi M, Amano N, Kurumatani N. Association between tooth loss and the development of mild memory impairment in the elderly: the Fujiwara-kyo Study. J Alzheimers Dis JAD 2015;44:777–86. doi: 10.3233/JAD-141665.

66. Iwasaki M, Yoshihara A, Kimura Y, Sato M, Wada T, Sakamoto R, et al. Longitudinal relationship of severe periodontitis with cognitive decline in older Japanese. J Periodontal Res 2016;51:681–8. doi: 10.1111/jre.12348.

67. Kaye EK, Valencia A, Baba N, Spiro A, Dietrich T, Garcia RI. Tooth loss and periodontal disease predict poor cognitive function in older men. J Am Geriatr Soc 2010;58:713–8. doi: 10.1111/j.1532-5415.2010.02788.x.

68. Sparks Stein P, Steffen MJ, Smith C, Jicha G, Ebersole JL, Abner E, et al. Serum antibodies to periodontal pathogens are a risk factor for Alzheimer’s disease. Alzheimers Dement J Alzheimers Assoc 2012;8:196–203. doi: 10.1016/j.jalz.2011.04.006.

69. ALFotawi R, Alzahrani S, Alhefdhi R, Altamimi A, Alfadhel A, Alshareef A, et al. The relation between teeth loss and cognitive decline among Saudi population in the city of Riyadh: A pilot study. Saudi Dent J 2020;32:232–41. doi: 10.1016/j.sdentj.2019.09.007.

70. Tiisanoja A, Syrjälä A-M, Tertsonen M, Komulainen K, Pesonen P, Knuuttila M, et al. Oral diseases and inflammatory burden and Alzheimer’s disease among subjects aged 75 years or older. Spec Care Dent Off Publ Am Assoc Hosp Dent Acad Dent Handicap Am Soc Geriatr Dent 2019;39:158–65. doi: 10.1111/scd.12357.

71. Rai B, Kaur J, Anand SC. Possible relationship between periodontitis and dementia in a North Indian old age population: a pilot study. Gerodontology 2012;29:e200-205. doi: 10.1111/j.1741-2358.2010.00441.x.
